# A study on the risk of fungal infection with tofacitinib (CP-690550), a novel oral agent for rheumatoid arthritis

**DOI:** 10.1038/s41598-017-07261-1

**Published:** 2017-07-28

**Authors:** Yong Chen, Fang-Yuan Gong, Zhen-Jun Li, Zheng Gong, Zhe Zhou, Shu-Yan Ma, Xiao-Ming Gao

**Affiliations:** 1 Institute of Biology and Medical Sciences (IBMS), School of Biology and Basic Medical Sciences, Soochow University, Suzhou, China; 2 Suzhou Kowloon Hospital, Shanghai Jiao Tong University School of Medicine, Suzhou, China

## Abstract

Tofacitinib (CP-690550), an oral *Janus* kinase inhibitor, has shown significant efficacy in the treatment of rheumatoid arthritis through blocking the signaling pathways of pro-inflammatory cytokines. However, recent evidence suggests that long-term tofacitinib treatment is associated with increased risk of infection (e.g. tuberculosis) in patients. In the present study, we illustrate that tofacitinib administration significantly reduced the survival rate of mice given lethal or sub-lethal dose challenge with Candida albicans. This was related to the ability of tofacitinib to reverse TNFα- and IFNγ-enhanced candidacidal activity of murine polymorph nuclear cells (PMNs) and also to suppress chemokine CXCL5 expression and PMN infiltration in the infected tissues of mice. More importantly, tofacitinib significantly antagonized the ability of TNFα, IFNγ and GM-CSF to boost human PMNs in phagocytosis and direct killing of *C. albicans in vitro*. It also down-regulated reactive oxygen production and neutrophil extracellular trap formation by human PMNs stimulated with yeast-derived β-glucans in the presence of TNFα, IFNγ or GM-CSF. Our data emphasizes a significantly increased risk for opportunistic fungal infection associated long-term tofacitinib treatment in humans, likely through antagonizing the PMN-boosting effect of pro-inflammatory cytokines.

## Introduction

In the past few years, several biologic therapies have been licensed to treat rheumatoid arthritis (RA) with significant efficacy. In 2012, tofacitinib (CP690550) became the first orally agent for RA treatment in the US, which expands the treatment options of patient which could not endure repeated injections for long time. However, recent evidence suggests that increased risk of opportunistic infection associated with long-term tofacitinib treatment is of considerable concern. Cases of tuberculosis (TB) infection were reported in several Phase II and III clinical trials^1, 2^. Winthrop *et al*. also observed occasional fungal and viral infections in addition to TB in tofacitinib-treated RA patients^3^. This is further supported by the work of Maiga and colleagues that tofacitinib administration in mice significantly increased their susceptibility to TB infection^4^.

Candida albicans represents a typical commensal microorganism, capable of causing opportunistic mucocutaneous candidiasis in immune compromised individuals. In the cases where the host immune system is severely compromised, *C. albicans* could rapidly proliferate and colonize various tissues of the body leading to a life-threatening disease^5^. Cytokine balance in the microenvironment plays a pivotal role against primary *C. albicans* infection. Th1 cytokines (e.g. IFN-γ, TNFα and IL-6) drive phagocytic cells into an enhanced candidacidal state, while Th2 cytokines (e.g. IL-4 and IL-10) deactivate phagocytic effector cells. More recent development suggests IL-17 (mainly produced by CD4^+^ Th17 cells and γδT lymphocytes) as a crucial player in anti-fungal defense, evidently through induction of cytokines and chemokines to promote PMN differentiation and migration^6^. Primary immunodeficiency patients with genetic mutations affecting IL-17 immunity are susceptive to chronic mucosal candidiasis (CMC)^7, 8^. Conti *et al*. further showed that IL-17-producing T lymphocytes and IL-17 receptor signaling are essential for mucosal host defense against oral candidiasis in mouse models^9^.

Tofacitinib is a selective inhibitor of the JAK family of kinases (with JAK3 as the main target, JAK1 and JAK2 also affected at higher concentration)^10, 11^, which would block intracellular signaling of several cytokine receptors including IL-2, IL-4, IL-7, IL-9, IL-15, IL-21 and IFNs. Because of JAK’s important role in the immune system, tofacitinib-mediated suppression may dampen immune responses involved in a broad spectrum of infectious diseases. Therefore in this study we focused on whether tofacitinib administration in mice would compromise their resistance to fungal infection and also whether tofacitinib directly inhibits the candidacidal activity of human neutrophils *in vitro*.

## Results

### Increased susceptibility to *C. albicans* infection in BALB/c mice under tofacitinib treatment

**Figure 1:**
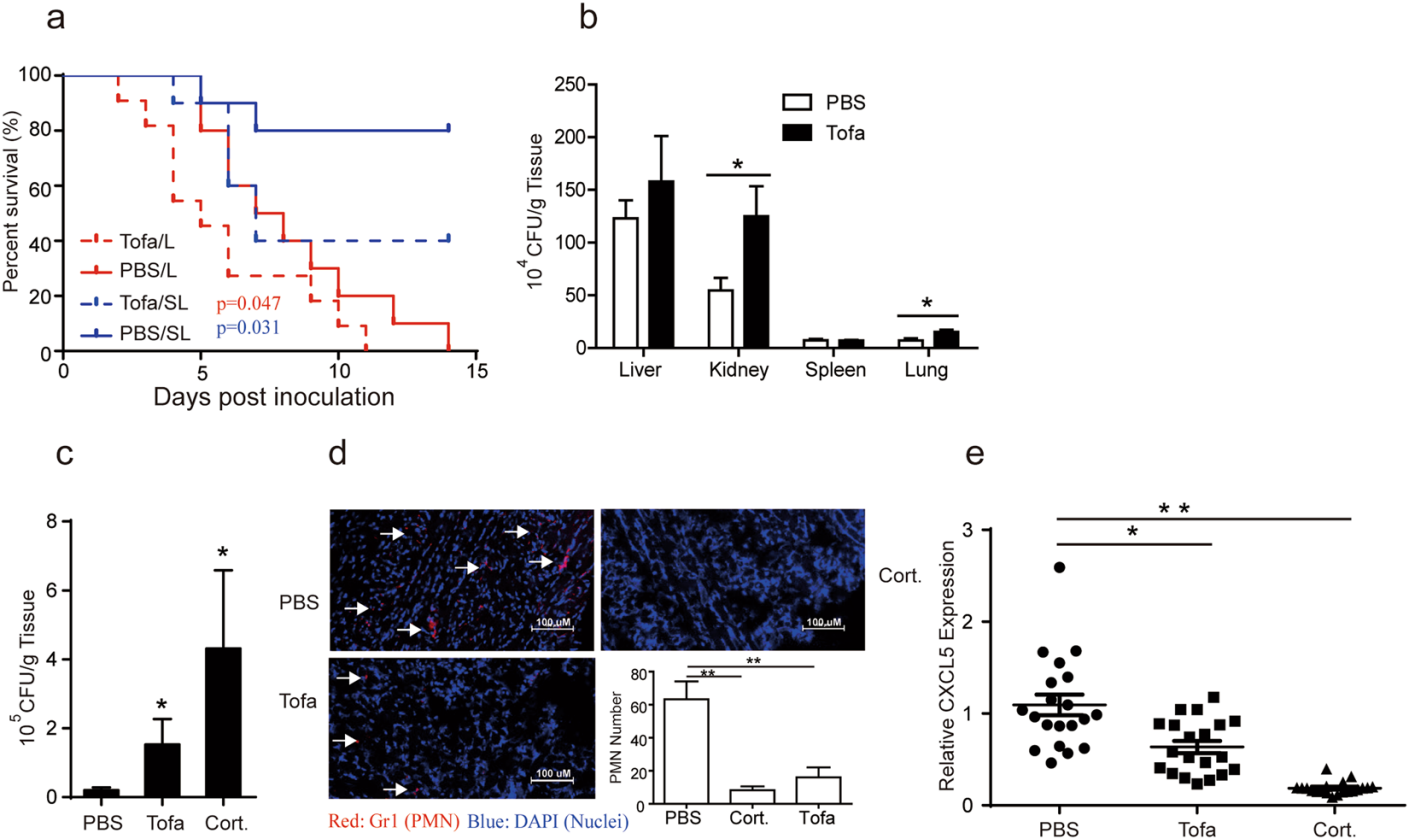
Tofacitinib increases the susceptibility of mice to *C. albicans* infection. (**a**) Survival curves of BALB/c mice (10 per group) after lethal (10^7^ CFU/mouse, i.v.), or sub lethethal (5 × 10^6^ CFU/mouse). *C. albicans* challenge followed by twice a day i.p. injections of tofacitinib at 15 mg/kg (*Tofa/L* and *Tofa/SL*, respectively), or PBS as control (*PBS/L* and *PBS/SL*, respectively). (**b**) Effect of tofacitnib on systemic *C. albicans* infection. BALB/c mice (5 per group) were given 2 i.p. injections (with 12 h intervals) of tofacitinib, or PBS as control, and then a lethal dose live *C. albicans* followed by 2 more injections of the same preparations on day 2. The animals were sacrificed 24 h post infection for assessment of *C. albicans* burdens in their livers, kidneys (both sides), spleens and lungs. The results are expressed as CFUs per gram tissue. (**c**) Effect of tofacitnib on mucosal *C. albicans* infection. BALB/c mice (8 per group) were given i.p. injections of tofacitinib (*Tofa*), or PBS, twice a day (with 12 h intervals) for 5 days. An additional group of mice was given 3 i.p. injections of cortisone acetate at 225 mg/kg (*Cort*.) once every other day beginning on Day -1. The mice were then challenged with live *C. albicans* in their oral cavity on day 0, followed by assessment of fungal load (CFUs/g tissue) in the tongues on day 5. (**d,e**) The effect of tofacitinib on PMN recruitment. Tongue tissue sections of mice were double stained with DAPI (blue) and Alexa Fluro 647 (red)-labeled Abs against mouse Gr1, followed by confocal laser-scanning macroscopic observation (arrows indicate Gr1-positive PMNs). Infiltrating PMNs were numerated for statistical comparison and the results are expressed as average number of PMNs per scope. The level of CXCL5 mRNA expression in the tongue tissues of mice was evaluated by Q-PCR. *p < 0.05, **p < 0.01. Results presented are representative of at least two independent experiments.

BALB/c mice were given i.v. a lethal (10^7^ CFU/mouse), or sub-lethal (5 × 10^6^ CFU/mouse), dose of live *C. albicans* followed by twice a day i.p. injections of tofacitinib (15 mg/kg), or PBS as control, for up to 14 days. Figure 1a shows that survival rates of the tofacitinib groups were significantly lower compared with the control group. To ascertain the effect of tofacitinib administration on proliferation of *C. albicans* during acute systemic infection, BALB/c mice were given 2 tofacitinib injections with 12 h intervals one day before and after *C. albicans* challenge (10^7^ CFU/mouse, i.v.), and fungal burden (CFU/g tissue) in the livers, kidneys, spleens and lungs of the infected animals were assessed 24 h post infection. Figure 1b shows that the number of viable *C. albicans* in kidneys and lungs of the tofacitinib group was significantly higher than that of the PBS control group. To assess whether tofacitinib could potentiate mucosal candidadasis, female BALB/c mice were given i.p. injections of either PBS, or tofacitinib (twice a day for 3 days), or cortisone acetate (once every other day beginning on Day -1) and then infected with viable *C. albicans* sublingually in oral cavity (10^7^ CFU/mouse) on Day 0. As shown in Fig. 1c, fungal burden in the oral cavities of the tofacitinib-treated animals (assessed on Day 5) was lower than that of the cortisone-treated group but significantly higher than that of the PBS-treated controls. Immunofluorescence staining of the infected mouse tongue tissue sections showed impaired neutrophil recruitment in the tofacitinib as well as the cortisone treatment groups (Fig. 1d). Our real-time PCR results further revealed significant suppression in the expression of CXCL5, a representative PMN chemokine, in the infected tongue tissue of the tofacitinib-treated group (Fig. 1e), which is in line with previous observation of reduced levels of chemokine expression and impaired neutrophil recruitment to the oral mucosa in oropharyngeal candidiasis (OPC) patients with immune (IL-17) deficiency^8^. Together these results confirm that tofacitinib administration renders mice significantly more susceptible to systemic as well as mucosal fungal infection.

### Inhibitory effect of tofacitinib on murine neutrophils *in vitro*

**Figure 2:**
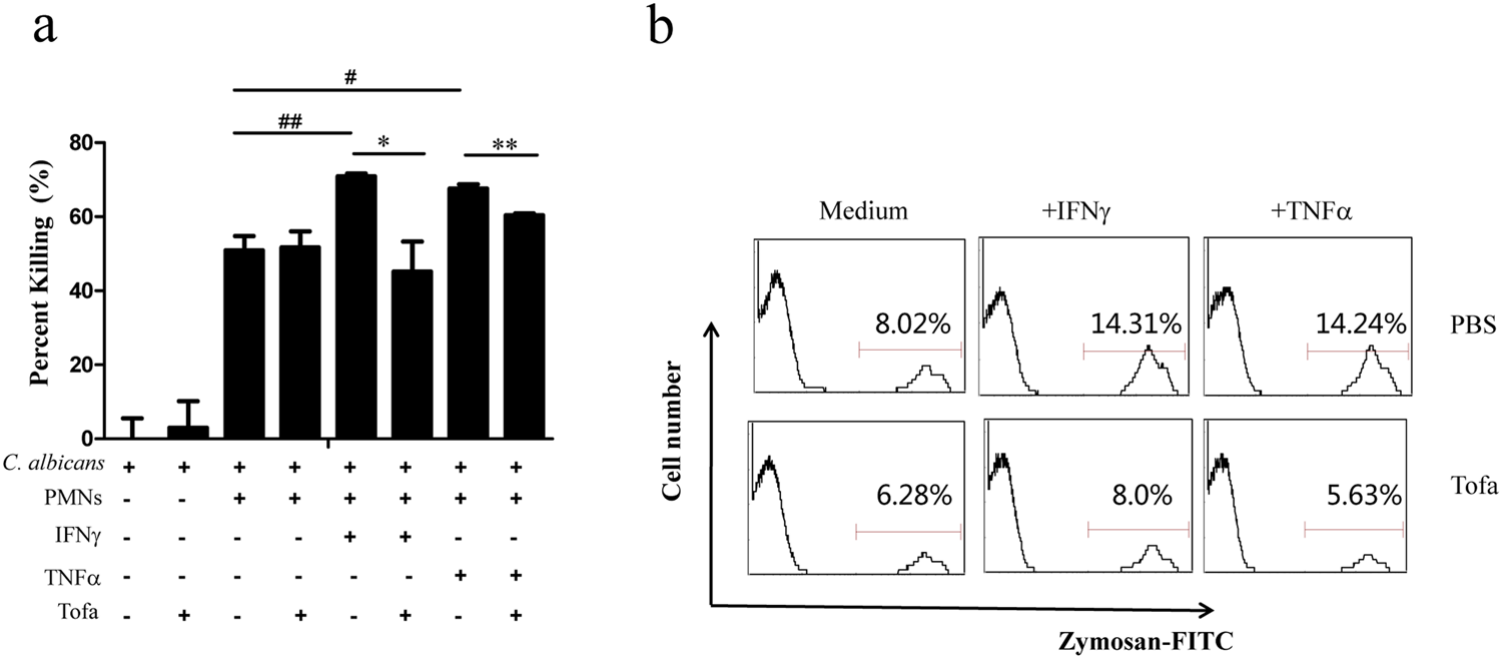
Inhibitory effect of tofacitinib on the candidacidal activity of mouse neutrophils. Freshly fractionated mouse neutrophils were pre-incubated with TNFα (200 U/ml) or IFNγ (10 ng/ml) in the presence, or absence, of tofacitinib (1 μM) for 2 h. Viable *C. albicans* cells were then added to the cultures for fungal killing assays, with *C. albicans* cultured alone, or in the presence of tofacitinib, as controls (**a**). The results are expressed as percent killing calculated as 100 × (total CFU-survived CFU)/total CFU. ^##^p < 0.01, ^#^p < 0.05, **p < 0.01, *p < 0.05. For phagocytosis analysis (**b**), PMNs were further treated with zymosan-FITC for 30 min followed by FACS analysis. Results presented are representative of three independent experiments.

It is well known that PMNs play vital roles in the defense against opportunistic fungal infection, as they are able to phagocytose and directly kill/digest captured pathogenic microorganisms. A likely pathway for tofacitinib to increase susceptibility of mice to fungal infection is through direct inhibition of murine PMNs. Figure 2a shows that PMNs isolated from the bone marrow of BALB/c mice were capable of killing C. *albicans in vitro*, but this was unaffected by 1 μM tofacitinib. Interestingly, the candidacidal activity of murine PMNs was significantly boosted (approximately 30% increase) by the addition of TNFα and IFNγ, that are well known to be important for the clearance of fungal infection *in vivo* ^5^. More importantly, such cytokine-mediated enhancement of PMN function was almost completely reversed in the presence of tofacitinib (Fig. 2a). β-glucans are major structural components of *C. albicans* and are recognized by phagocytic cells as pattern molecules^12^. FITC-labeled zymosan particles (derived from yeast cell walls) were employed to quantitatively assess the phagocytosis activity of PMNs in FACS analysis. Figure 2b shows that the capture/phagocytosis of zymosan by TNFα- and IFNγ-boosted PMNs was also reversed by tofacitinib. Thus, tofacitinib is an antagonizing agent against proinflammatory cytokines in PMN activation, which could help explaining the fungal infection potentiating effect of tofacitinib *in vivo*.

### Inhibitory effect of tofacitinib on the phagocytosis and candidacidal activities of human PMNs

**Figure 3:**
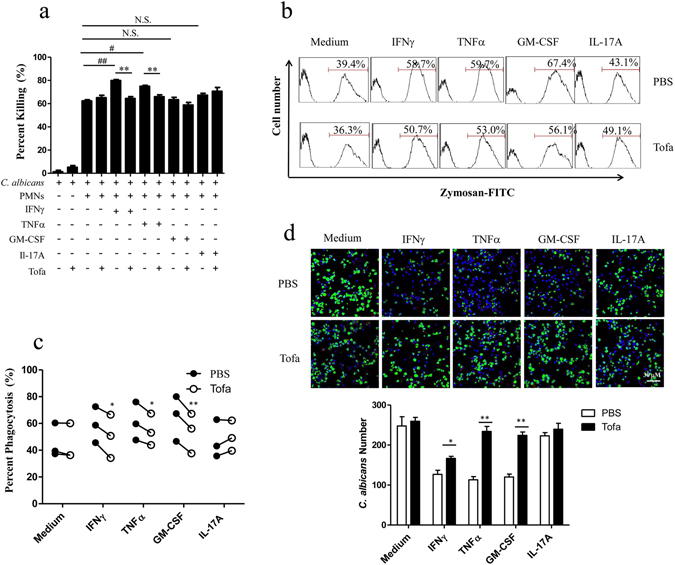
Inhibitory effect of tofacitinib on the candidacidal activity of human PMNs. Freshly isolated human PMNs were pre-treated, in triplicate wells, with IFNγ (10 ng/ml), TNFα (200 U/ml), GM-CSF (10 ng/ml) or IL-17A (10 ng/ml) in the presence, or absence, of tofacitinib (1 μM). Viable *C. albicans* cells were then added to the cultures for fungal killing assays, with *C. albicans* cultured alone as total CFU control (**a**). The results are expressed as percent killing calculated as 100 × (total CFU-survived CFU)/total CFU. ^##^p < 0.001; ^#^p < 0.05; **p < 0.01. For phagocytosis analysis, cytokine-treated PMNs (with or without tofacitinib) were fed with zymosan-FITC for 30 minutes followed by FACS analysis. Representative results using blood samples from one donor are shown as histograms, fluorescence positive peaks (indicated by red bars) represent FITC-zymosan-containing cells (**b**). Repeating results using samples from 3 unrelated donors were individually converted into percent phagocytosis (fluorescence positive cells) for statistical comparison between groups treated with PBS (filled circle) or tofacitinib (open circle) (**c)**. Each pair of filled and open circles represents data from one donor. *p < 0.05, **p < 0.01. (**d**) For further visualization of the inhibitory effect of tofacitinib on fungal digestion by PMNs, human PMNs were treated as above, then incubated with FITC-labeled opsonized *C. albicans* for 4 h, followed by 4% PFA fixation and DAPI staining. Images were acquired by confocal microscopy and undigested *C. albicans* in each group were numerated for statistical comparison. The results are expressed as average number of *C. albicans* per scope. *p < 0.05, **p < 0.01.

We next addressed the question whether tofacitinib could inhibit the phagocytosis and candidacidal activity on human PMNs under stimulation of TNFα and IFNγ, and obtained results similar to that using mouse cells (Fig. 3a–c). Given that IL-17A and GM-CSF also exhibit anti-fungal protective effect *in vivo* ^6, 7, 13^, they were also analyzed in parallel experiments. In our hands, however, neither of them was able to directly boost the candidacidal activity of human PMNs *in vitro* (Fig. 3a). Interestingly, GM-CSF, but not IL-17A, significantly enhanced zymosan phagocytosis by human PMNs in a tofacitinib-sensitive manner (Fig. 3b and c).

Successful intracellular killing/digestion of fluorescence-labeled yeast particles by phagocytes leads to significant fluorescence quenching, demonstrable by laser-scanning confocal microscopy. In order to investigate if tofacitinib could inhibit intracellular killing/digestion of *C. albicans* by human PMNs, freshly fractionated human PMNs were fed with FITC-labeled *C. albicans*, washed and then incubated in culture medium containing either IFNγ, or TNFα, or GM-CSF, or IL-17A at 37 °C for 4 h in the presence, or absence, of tofacitinib. Figure 3d shows that TNFα, IFNγ and GM-CSF, but not IL-17A, substantially enhanced the candidacidal activity which was reversible by tofacitinib.

### Tofacitinib modulates anti-fungal responses of human PMNs

**Figure 4:**
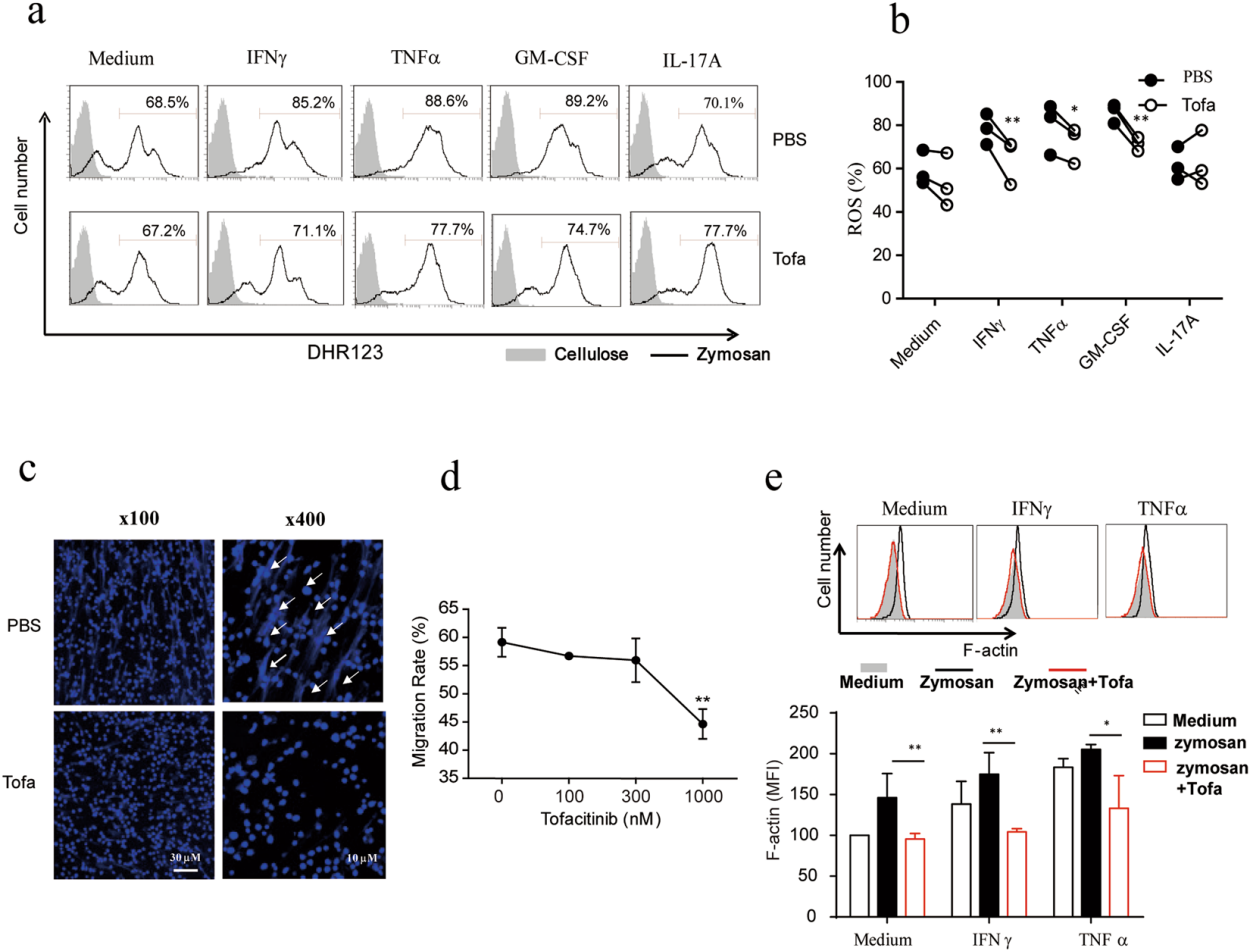
Effect of tofacitinib on the activation and function of human PMNs. (**a**,**b**) Freshly isolated human PMNs were treated with IFNγ (10 ng/ml), TNFα (200 U/ml), GM-CSF (10 ng/ml) or IL-17A (10 ng/ml) in the presence, or absence, of tofacitinib (1 μM) for 2 h, followed by DHR123 loading and stimulation with zymosan (50 μg/ml). ROS production was evaluated by detecting fluorescence-converted DHR123 in the cells using a FACS machine. Representative results using blood samples from one donor are shown as histograms, peaks representing ROS-positive cells are indicated by red bars (**a**). Repeating results using samples from 3 unrelated donors were individually converted into percent ROS-positive cells for statistical comparison between groups treated with PBS (filled circle) or tofacitinib (open circle) (**b)**. Each pair of filled and open circles represents data from one donor. *p < 0.05, **p < 0.01. For NET formation assay (**c**), freshly isolated human PMNs were stimulated with with zymosan (50 μg/ml) for 4 h, followed by followed by 4% PFA fixation and DAPI staining. Images were acquired by confocal microscopy at 100x and 400 magnifications. Arrows indicate traps (NETs) formed. For leukocyte migration assay (**d**), human PMNs were incubated with different concentrations of tofaicitinib for 2 h in the top chamber of a transwell system, then fMLP (100 nM) was in the bottom chamber and allowed a further 2 h incubation. After fixation and DAPI staining steps, migrated cells were counted, and the results are shown as ratio of migration, calculated as 100 × (migrated cell number/starting cell number). **p < 0.01. For F-actin formation analysis (**e**), human PMNs were pre-incubated with IFNγ or TNFα in the presence, or absence, of tofacitinib (1 μM), followed by stimulation with zymosan for 4 h, untreated cells were included as control. Aggregated F-actin in the cells was stained by fluorescence labeled phalloidin and visualized by FACS analysis. Representative histograms are shown in the upper panels and the statistic results of three independent experiments (expressed by mean fluorescence intensity of peaks) are given in the lower panel. *p < 0.05, **p < 0.01.

PMN phagocytosis of yeast or zymosan particles leads to oxidative burst and generation of reactive oxygen species (ROS), detectable with DHR123 which displays green fluorescence after reaction with ROS^14^, and formation of neutrophil-derived extracellular traps (NETs, combination of DNA fibers and granular enzymes), which could immobilize extracellular organisms providing the host with an effective extracellular antifungal defense^15^. Figure 4a and b show that zymosan-induced ROS generation by human PMNs was augmented by IFNγ, TNFα and GM-CSF, but readily reversible by tofacitinib. Yeast- or zymosan-stimulated PMNs showed characteristic features of nectotic cells and formed fiber-like DNA lattice structure protruding from cells, which was also inhibited by tofacitinib (Fig. 4c). Furthermore, tofacitinib significantly inhibited the migration of PMNs towards fMLP, a prototypic microbial chemotract agent, in leukocyte migration assays (Fig. 4d). The reduced F-actin polymerization on the surface of human PMNs in the presence of tofacitinib, detected by fluorescence-labeled phalloidin, provides additional evidence for the inhibitory effect of tofacitinib on the function of PMNs (Fig. 4f).

### Inhibitory effect of tofacitinib on human monocytes

**Figure 5:**
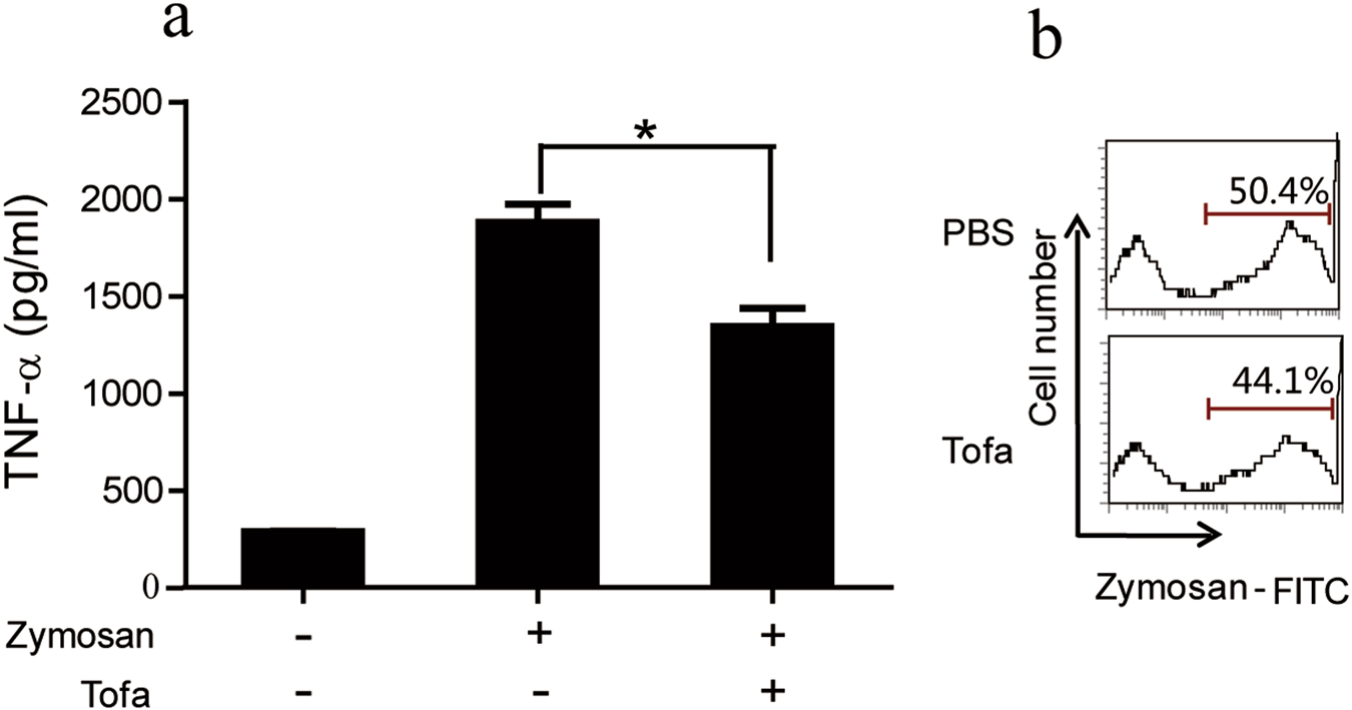
Effects of Tofacitinib on *C. albicans*–induced PBMC and monocyte responses. (**a**) Freshly prepared human PBMCs were stimulated with zymosan in the presence, or absence, of tofacitinib (1 μM) for 24 h, and then TNFα in the supernatant quantitated by ELISA. *p < 0.05. (**b**) CD14^+^ monocytes, fractionated from human PBMC, were treated with tofacitinib or PBS for 2 h and then with FITC-labeled zymosan for 1 h, followed by FACS analysis. Representative histograms are shown in the left panels with zymosan-containing positive peaks indicated by red bars. Repeating results using samples from 3 unrelated donors were converted into phagocytosis (fluorescence positive cells) for statistical comparison between groups treated with PBS (filled circle) or tofacitinib (open circle). **p < 0.01.

Besides PMNs, monocytes/macrophages represent another indispensable arm of the innate immune system against pathogenic infections. They are able not only to phagocytose and digest pathogens but also secret pro-inflammatory cytokines to enhance killing activity of PMNs. Figure 5a shows that tofacitinib inhibited TNFα release by human PBMC stimulated with zymosan. Tofacitinib also directly inhibited the phagocytosis of FITC-zymosan by fractionated human monocytes (Fig. 5b).

## Disscussions

RA is an autoimmune inflammatory joint disease characterized by systemic, destructive, and progressive inflammatory synovitis, which needs long-term treatment with immunosuppressive drugs. A major concern for such agents is increased risk of opportunistic infection in patients due to compromised immunity. The blood level of tofacitinib during RA treatment could reach 1 μM as evidenced in pharmacokinetics studies^16^. Our results show that, in this dose range, tofacitinib does not affect the candidacidal activity of PMNs per se, but could reverse pro-inflammatory cytokine-boosted candidacidal activity of the cells.

Molecular mechanisms of anti-fungal immune responses have been extensively investigated in the past. It is clear that protective anti-candidal responses require concerted actions of various cytokines, such as IFN-γ, IL-6, TNF-α, IL-12 and IL17 that are produced by different types of immune cells including macrophages, NK cells, Th1 and Th17^5, 6, 17–21^. Candidacidal ability of PMNs or macrophages can be significantly boosted by exposure to pro-inflammatory cytokines such as IFNγ and TNFα^5^, which is confirmed in our study.

Ample evidence suggests an important role of IFN-γ in host defense against fungal infection^21^. For example, Lilic *et al*. have reported negative correlation between IFN-γ levels and CMC in humans^22^. Balish and colleagues have shown that mice deficient in IFN-γ or IFN-γ receptor are significantly more susceptible than wild-type mice to systemic *C. albicans* infection^23^, although this is contradicted by a more recent report of Farah *et al*. that IFN-γ-knockout mice are not more susceptible to mucosal candidiasis^24^. Administration of IFN-γ to mice infected with *C. albicans* showed a beneficial effect on the outcome of the infection^25^. However, the role of IFN-γ in anti-fungal immunity in humans appears to be much more complicated than in mouse models. Liu etc reported that patients with hyperactive STAT1 tend to suffer from CMC^26^, implying over-activation by cytokines such as IFN-γ may disrupt coordinated response of the immune system against fungal infection. Recombinant IFN-γ is widely used for the treatment of chronic granulomatous disease (a genetic disorder characterized by recurrent bacterial and fungal infections and tissue granuloma formation), however, invasive fungal infection has remained a persistent problem in these patients^27^.

IFN-γ is a type II IFN with JAK1-STAT1 as its major signaling pathway. Although tofacitinib was initially thought to be a JAK3-specific inhibitor, more recent data using cell-free assay systems indicates that in higher concentrations it could also inhibit JAK1 and JAK2^28^. Recent findings have demonstrated that type I IFNs have an even more significant role than IFN-γ in host defense against *C*. *albicans* in humans^29^. Given that JAK1-STAT1/2 is an essential and specific positive effector of type I IFN signaling, tofacitinib could also exert its inhibitory effect on anti-fungal immunity via interfering the function of type I IFNs.

TNF-α appears to be essential for the successful control of early fungal infection, as it is able to recruit neutrophils to the infection sites and enhance phagocytic activity of phagocytes^30^. TNF-α deficiencies render mice more susceptible to *C. albicans* infection^19^. There are also case reports for candidiasis in patients on TNF-α antagonist therapy^31–33^. However, TNFα does not signal directly by the JAK-STAT pathway, raising important questions regarding the molecular mechanisms for a TNFα antagonizing role of tofacitinib. Rosengren *et al*. and Yarilina *et al*. have recently shown that TNF-α induces STAT1 phosphorylation and up-regulates STAT1 targeted genes in human macrophages and fibroblast-like synoviocytes via a TNFα-IFNα-JAK-STAT1 autocrine loop^28, 34^. The possibility for a similar activation mechanism in PMNs and macrophages can be anticipated. In addition, it is also evident that JAK inhibitors could inhibit TNFα responses through suppression of late phase NF-κB signaling which was related to JAKs^34^.

GM-CSF is a pleiotropic cytokine regulating the survival, proliferation and differentiation of myeloid cells. It has been shown that GM-CSF substantially enhances the antifungal activity of PMNs *in vivo* ^35^. GM-CSF–deficient mice are susceptible to a wide range of pathogens including *C. albicans* ^13^. GM-CSF could also enhance phagocytosis and ROS generation of PMNs for clearance of *C. albicans* ^35^. It is well known that GM-CSF activates the JAK2-STAT3 signaling pathway, which could be inhibited by tofacitinib at 1 μM concentration. The work by Pena *et al*. showed that JAK2 is a principle JAK required for anti-fungal immunity^36^. We also tested ruxolitinib, a selective JAK2 inhibitor, in parallel experiments with similar results (data not shown), which is in line with a recent report of Tsirigotis *et al*. showing inhibitory effect of ruxolinitib (administered prior to infection as in our study) on anti-fungal immunity in mice^37^.

Recent results suggest IL-17 as a key player in anti-fungal defense in humans^6–9, 38^. In our study, however, IL-17 did not boost candidacidal acitivity, phagocytosis ability and ROS generation of human PMNs *in vitro*. This is perhaps not surprising because the major effect of IL-17 in immune response against extracellular bacterial or fungal pathogens is to help the recruitment of neutrophils by induction of appropriate chemokines^6, 9, 38^. Interestingly, tofacitinib suppressed CXCL5 expression as well as infiltration of neutrophils in the tongues of *C. albicans* infected mice (Fig. 1d and e), which is further supported by our unpublished observation that IL-17-induced chemokine production in a human cell line 293 T was susceptible to tofacitinib inhibition (data not shown). Molecular mechanisms for tofacitinib modulation of IL-17 function are not entirely clear. Subramaniam etc documented that various JAK isotypes, such as JAK-1, -2, -3, and Tyk-2, can interact with IL-17RA on binding to its ligand in cell lines^39^.

Taken together, our data demonstrates that the risk of fungal infection should be monitored as well as other opportunistic infections after long-term tofacitinib use. Combination with anti-fungi drugs may be necessary in patients showing signs of increased susceptibility to infection.

## Materials and Methods

### Chemical reagents

RPMI-1640 medium and Dulbecco’s modified Eagle’s medium (DMEM), Martin medium and penicillin-streptomycin (100×) were obtained from Invitrogen (Calsbad, CA, USA). Fetal bovine serum was from Hyclone (Logan, UT, USA) and was heat-inactivated at 56 °C for 30 min prior to use. Polymorphprep was obtained from Axis-Shield (Scotland,UK). Percoll was from Pharmacia (Pittsburgh, PA,USA). Zymosan, zymosan-FITC, PMA, cellulose, DHR123 and fMLP were purchased from Sigma (St. Louis, MO, USA). Tofacitinib (CP-690550) was purchased from Selleck (Houston, TX, USA). Cytokines including TNFα, IFNγ, GM-CSF and IL-17A were purchased from Pepro-Tech (Rocky Hill, NJ, USA).

### Donors, mice and cells

Blood samples were obtained from healthy non-pregnant donors of 23–35 years of age, and informed, written consent was obtained from each subject. The methods were carried out in accordance with the guidelines of Soochow University. Female BALB/c mice of 10–12 weeks of age were purchased from Nanjing Model Animal Institute, Jiangsu, China. The use of human cells and experimental animals (mice) as well as all experimental protocols were approved by the Ethic Committee of Soochow University. All animal experiments were performed according to the guidelines for the Care and Use of Laboratory Animals (Ministry of Health, China, 1998).

Human peripheral blood PMNs and monocytes were collected as previously described^40^. In brief, peripheral blood mononuclear cells (PBMC) were isolated immediately after polymorphprep gradient centrifugation (500 g, 30 min, room temperature). Monocytes were further enriched by anti-CD14 monoclonal antibody-conjugated microbeads (Miltenyi Biotec, Germany) according to the manufacturer’s instruction.

Mouse neutrophils were isolated according to the method of Furze *et al*.^41, 42^ with modification. Briefly, bone marrow cells of BALB/c mice were centrifuged in a discontinuous Percoll gradient of 52%, 62% and 74%, followed by collection of the cells in the 62%/74% interface.

### *C. albicans* infection of mice

*C. albicans*, obtained from the clinical laboratory of Suzhou municipal hospital, were cultured in Martin Medium at 37 °C to late exponential growth phase. The CFU of *C. albicans* was determined by enumerating clone numbers 24 h after different concentration of *C. albicans* seeded on agar plate. For systemic infection experiment, BALB/c mice were i.v. injected with a lethal (10^7^ CFU/mouse),or sub-lethal (5 × 10^6^ CFU/mouse), dose of *C. albicans* followed by either survival rate monitoring/recording every 12 h for up to 2 weeks or sacrifice 24 h post infection for assessment of fungal burdens in organs. Tofacitinib (15 mg/kg), and PBS as control, was intraperitoneal injected twice a day after the infection. For corticosteroids control, mice were i.p. injected with cortisone acetate 225 mg/kg (Sigma-Aldrich) on days -1, 1, and 3. Organs including liver, lung, spleen and both kidneys from the sacrificed animals were homogenized by mechanical disruption in PBS. Serial dilutions were plated on Sabouraud agar and incubated at 37 °C for 24 h. Colonies were counted and expressed as CFU per organ. Oral mucosal infection was done by placing saturated cotton pads with *C. albicans* (10^7^ CFU/mouse) in the oral cavity of mice and keeping the mice in a supine position for about 1.5 h. The tongue tissues were removed 5 days post inoculation. After weight recording, the tissue samples were separately homogenized in sterile saline and then placed on Martin Medium agar after proper dilution. The fungal burden was evaluated by counting CFU after growth at 37 °C for 24 h.

### Fungal killing assays

PMNs freshly isolated from human blood or mouse bone marrow were pre-treated with cytokines (10 ng/ml IFNγ, 200 U/ml TNFα, 10 ng/ml GM-CSF or 10 ng/ml IL-17A) in the presence, of absence, of tofacitinib (1 μM) for 2hrs at 37 °C. The cells were then infected with non-opsonized (in the case of mouse cells) or opsonized (in the case of human cells) live *C. albicans* (at a ratio of 2 CFU per cell) for 4 hrs in an incubator at 37 °C with gentle shaking every 10 minutes. The same number of viable *C. albicans* cells was cultured in medium alone as total CFU control. Finally, the cells were collected by centrifugation at 2500 g and the pellet re-suspended in PBS containing 0.1% Triton X-100, followed by enumeration of viable *C. albicans* cells on agar plates. Percent killing of the fungal cells by PMNs was calculated as 100 × (total CFU-survived CFU)/total CFU.

### Confocal microscopy

PMNs (2 × 10^5^) were allowed to uptake FITC-labeled *C. albicans* (4 × 10^5^) in 200 μl culture medium in Poly-L-Lysinecoated 8 well chamber slide system (Nunc) for 4 h, and then fixed with 4% paraformaldehyde (PFA) followed by staining with PE-labeled anti-CD11b Ab for cell membrane and DAPI for the nuclei. The specimens were observed using a laser-scanning confocal microscope (A1, Nikon, Japan) though red, violate and green channels. Frozen tongue sections were stained with Alexa Fluro 647 labeled anti-Gr1 and DAPI and analyzed at 20 × T using a laser-scanning confocal microscope (A1, Nikon, Japan).

### Phagocytosis assays

Neutrophils or monocytes (2 × 10^5^ cells/tube) were pre-treated with IFNγ (10 ng/ml), TNFα (200 U/ml), GM-CSF (10 ng/ml) or IL-17A (10 ng/ml), in the presence, or absence, of tofacitinib (1 μM) in RMPI-1640 medium containing 10% FCS for 2 h at 37 °C.The cells were collected by centrifugation and re-suspended in 100 μl medium containing human serum-opsonized FITC-labeled zymosan (8 μg), and further incubated at 37 °C for 1 h with continuous shaking to ensure moderate blending state. The phagocytosis process was stopped by adding 4% PFA to the culture, and the cells were analyzed using an Attune NxT Flow Cytometer (Life Technology, CA, USA) for phagocytosis fluorescence-labeled fungal cells by PMNs.

### ROS production assay

Neutrophils (2 × 10^5^ cells/tube) were pre-treated with IFNγ (10 ng/ml), TNFα (200 U/ml), GM-CSF (10 ng/ml) or IL-17A (10 ng/ml), in the presence, or absence, of tofacitinib (1 μM) in RMPI-1640 medium containing 10% FCS for 2 h at 37 °C.The cells were then loaded with 1 μM DHR123, are duced form of rhodamine 123, which shows fluorescence when oxidized by oxidative species or by cellular redox systems to the fluorescent rhodamine, for 30 minutes, after wash, stimulated with zymosan (50 μg/ml) for another 60 min with continuous rotation at 37 °C. Reactions were stopped by placing the tubes on ice prior to FACS analysis using an Attune NxT Flow Cytometer.

### NET Formation and F-actin assay

Freshly isolated PMNs (10^5^ cells), seeded in 200 μl medium on Poly-L-Lysine coated 8-well chamber slide system (Nunc), were stimulated with 50 μg/ml zymosan for 4 h at 37 °C and then fixed with 4% PFA for 30 min. For visualization of neutrophil-derived extracellular traps (NETs), the cells were stained with DAPI and examined by laser scanning confocal microscopy.

For F-actin assay, freshly isolated PMNs were stimulated with 50 μg/ml zymosan for 4 h at 37 °C. After 4% PFA fixation, the cells were further treated with 0.1% Triton-100 for 10 min. Aggregated actin in the cells was detected with Alexa Fluor 488 labeled phalloidin, a F-actin tracker, and FACS analysis.

### PMN migration assay

PMN migration assay was essentially the same as that of Filippi *et al*.^42^. In brief, 5 μm pore transwell filters (Costar) with fibrinogen coated at the bottom were placed on 24 well cell culture plate. PMNs were pretreated with different concentration of tofacitinib for 2 hours and placed in the top chamber. 100 nM fMLP was placed in the bottom chamber as chemotract at 37 °C for 2 hrs. Neutrophils that migrated across the filter were counted after DAPI staining. Percent migration was expressed as 100 × (migrated cell number/starting cell number).

### Q-PCR

Total cellular RNA was extracted from frozen tongue tissues or pellets of 293 T cells with HP Total RNA Kit (Omega) according to the manufacturer’s instruction. One microgram of total cellular RNA was used as template for cDNA synthesis with a Reverse Transcriptase M-MLV (Takara, JP). Relative quantification of indicated genes was determined by real-time PCR with SYBR Green (Takara, JP) probe normalized to HPRT on a Step one PCR System (Applied Biosystems, CA, USA). The following primers were employed for specific amplification of murine CXCL5 encoding sequence: *F-TGGGCAGTGACAAAAAGAAAGC*, *R-TTCACTGGGGTCAGAGTCCT*.

### Statistical analysis

All statistical analyses were performed with GraphPad Prism Software version 4. Unpaired Student t tests were used to compare differences between the groups. The log-rank test and the Mann–Whitney U test were used to analyze survival curves and fungal counts, respectively. Error bars represent the SEM of the mean. A *p* value < 0.05 was considered statistically significant.
